# Supplementary material for: Large-scale gene expression alterations introduced by structural variation drive morphotype diversification in Brassica oleracea
Source: Nat Genet. 2024 Feb 13;56(3):517–29. doi: 10.1038/s41588-024-01655-4 (PMC10937405; doi:10.1038/s41588-024-01655-4)
Supplement: Supplementary file 1 — Supplementary Notes 1–5 and Supplementary Figs. 1–12. [file 41588_2024_1655_MOESM1_ESM.pdf]

nature genetics

Article

https://doi.org/10.1038/s41588-024-01655-4

Large-scale gene expression alterations introduced by structural variation drive morphotype diversification in *Brassica oleracea*

In the format provided by the authors and unedited

Table of Contents

Supplementary Note 1: Materials and Methods...2

Supplementary Note 2: High Quality Genome Assembly of Representative Morphotypes 5

Supplementary Note 3: Homoeologous Gene Retention Variation...7

Supplementary Note 4: SVs Introduce Expression Variation in Numerous Genes...................9

Supplementary Note 5: Expression Alterations by SVs Associate with Morphotypes............10

Supplementary Tables...12

Supplementary Figures...19

Source Data Supplementary Fig. 9 31

Supplementary References...32

Supplementary Note 1: Materials and Methods

1.1 Library construction and sequencing

A total of 22 *B. oleracea* accessions were selected for *de novo* genome assembly in this study, among which 17 accessions were sequenced by Institute of Vegetables and Flowers, Chinese Academy of Agricultural Sciences (IVF-CAAS, Beijing, China) and five accessions (T06, T24, T25, T26 and T27) were sequenced by Plant Breeding, Wageningen University & Research (PBR-WUR, Wageningen, the Netherlands). The 17 and five accessions were grown in the greenhouse of IVF-CAAS in 2020 and in the Unifarm of WUR in 2019, respectively. For the 17 IVF-CAAS accessions, Illumina libraries were constructed using the TruSeq Nano DNA HT Sample Preparation Kit (Illumina, CA, USA). Oxford Nanopore libraries were constructed using the SQK-LSK109 Ligation Sequencing Kit. PacBio SMRT libraries were constructed based on the protocol of SMRT bell library construction (https://www.pacb.com/support/documentation/). Sequencing of these constructed libraries were performed on the Illumina HiSeq 2000 platform, and PacBio Sequel or Oxford Nanopore MinION or PromethION platforms. Hi-C libraries were constructed for 16 out of 17 accessions using a previously described approach [1], which were then sequenced on the Illumina HiSeq 2000 platform.

For the five PBR-WUR accessions, young leaves were collected and high molecular weight (HMW) DNA was extracted for each plant following a previously established protocol [2]. The SQK- LSK109 Ligation Sequencing kit (Oxford Nanopore Technologies; Oxford, UK) was used for library constructions according to manufacturer’s instructions. Long-read sequencing data were generated using the Oxford Nanopore GridION platform and a run-time of 48 hr. In addition, genomic DNA was extracted from these young leaves using a cetyltrimethylammonium bromide (CTAB) method [3]. Illumina libraries with ~450 bp and ~600 bp insertion sizes were constructed and the resulting libraries were sequenced on Illumina Hiseq 2500 (~450 bp libraries) and X10 (~600 bp libraries) platforms. Moreover, high Molecular Weight

plant DNA was extracted from fresh tissue of the five morphotypes using the Bionano Prep™ Plant Tissue DNA Isolation Kit. The Direct Label and Stain (DLS) technology, together with Bionano Saphyr platform, were used for generation of optical mapping data. DLS labeling was performed with 750 ng DNA using the Direct Labeling and Staining Kit (Bionano Genomics Catalog 80005) following manufacturer's recommendations. The loading of labeled DNA onto Saphyr chip and running of the Bionano Genomics Saphyr System were all performed according to the Saphyr System User Guide (https://bionanogenomics.com/support-page/saphyr-system/).

The mRNA-seq data were generated to aid in genome annotation. For 16 of the 17 IVF-CAAS accessions (**Supplementary Table 17**), five tissues were collected, including root, stem, leaf, flower, and seed pods. Total RNA was isolated using the RNAprep Pure Plant Kit (TIANGEN Biotech, Beijing, China), following which RNA-seq library was constructed using a reported method [4] and sequenced on the NovaSeq platform. For each of the five PBR-WUR accessions, whole young seedling and different tissues including leaves, meristems, curds, stems, and flowers were pooled in one mRNA-seq library. These five mRNA-seq libraries were sequenced by the Illumina NovaSeq platform with 150 bp paired-end reads. Besides, we performed mRNA-seq of leaf for 223 out of the 415 accessions.

Additionally, whole genome bisulfite sequencing (WGBS) was performed for 16 out of the 17 IVF-CAAS accessions (**Supplementary Table 18**). Briefly, 2 ug of genomic DNA was purified by CleanNGS-R beads (Vdo Biotech, China) to eliminate contamination of genomic DNA. Then, 1 µg of purified genomic DNA spiked with 20 ng unmethylated Lambda DNA was fragmented by sonication to a mean size of approximately 200-500 bp. After bisulfite treatment (EZ DNA Methylation-GoldTM Kit, Zymo Research, Cat. No. D5005), the DNA was amplified with 13 cycles of PCR using Illumina 8 bp dual index primers. The constructed WGBS libraries were then analyzed by Agilent 2100 Bioanalyzer. The clustering of the index-coded samples was performed on a cBot Cluster Generation System using Illumina Novaseq 6000 S4 Reagent Kit according to the manufacturer's instructions. After cluster generation, the

DNA libraries were sequenced on Illumina NovaSeq 6000 platform and 150 bp paired-end reads were generated.

1.2 Luciferase report experiment

Primers used for luciferase report experiment:

Mini-MYBproF:

GGTCGACGGTATCGATAAGCTTACTGCAGCATGGAACTACG

Mini-MYBproR:

GGAAGGGTCTTGTCTAGAACTAGTGTGGTTTGAAAAGAAGTCGCC

Mini-MYBSVF:

CTATAGGGCGAATTGGGTACCTCAGACCATCAGCATTGGTAAACC

Mini-MYBSVR:

TTGTCTAGAACTAGTGGATCCGAGCATCAGCATTGGAGGATTATAAC

Supplementary Note 2: High Quality Genome Assembly of Representative Morphotypes

To construct a pan-genome that encompasses the full range of genetic diversity in *B. oleracea*, we analyzed the resequencing data of 704 globally distributed *B. oleracea* accessions covering all different morphotypes and their wild relatives (**Supplementary Tables 1** and **2**). Phylogenetic analysis using SNPs classified the 704 accessions into three main groups (**Fig. 1a**). The first group included accessions of wild *B. oleracea* and different kales. The distal cluster of the second group was composed of broccoli and cauliflower with separated branches for Chinese kale, Tronchuda, and kohlrabi (hereafter referred to as 'AIL', abbreviation for arrested inflorescence lineage and closely related morphotypes). The third group consisted of the leafy head cabbage accessions, plus ornamental kale and Brussels sprouts (hereafter referred to as 'LHL', abbreviation for leafy head lineage and closely related morphotypes).

Based on the phylogeny and morphotype diversity, we selected 22 representative accessions for *de novo* genome assembly (**Table 1**). These accessions were sequenced using PacBio or Nanopore long-read sequencing technologies with an average coverage of 133×, and Illumina short-read sequencing technology with an average coverage of 250× (**Supplementary Tables 3-6**). In addition, we generated an average coverage of 267× chromosome conformation capture (Hi-C) data for 16 IVF-CAAS accessions and an average coverage of 212× BioNano data for five WUR accessions. The long reads were *de novo* assembled into contigs that were further polished using both long- and short-reads (**Methods**). Seven genomes with relatively high heterozygosity (> 0.3%) (**Supplementary Table 7**) were purged to remove potential redundant homologous sequences.

A total of 53.5%–58.5% sequences in 27 (our 22 new genomes and five previously reported genomes) [5-8] *B. oleracea* genomes were annotated as TEs, compared to 47.1% in the sister species *Brassica rapa* [9]. The long terminal repeat retrotransposons (LTR-RTs) were the most abundant TEs, representing 26.1% to 31.2% of sequences in the 27 genomes, with *Copia* and *Gypsy* accounting for an average of 39.2% and 44.0% of all

LTR-RTs, respectively. We further identified a range of 4,703 to 6,253 full-length LTR-RTs (fl-LTRs) in these genomes (**Supplementary Table 9**). Recently inserted fl-LTRs were enriched in centromeric regions (**Fig. 1c**). On average, 2,896 and 1,722 full-length *Copia* and *Gypsy* LTRs, respectively, were identified in these genomes. We revealed continuous expansion of *Copia* and *Gypsy* in all these genomes since four MYA (**Fig. 1d**). The number of *Copia* insertions has always been higher than that of *Gypsy*, with a recent (< 0.20 MYA) increase of *Copia* insertions being almost twice that of *Gypsy*. Additionally, *Copia* TEs were clustered into more and larger groups than *Gypsy* based on sequence similarity (**Fig. 1d**). Together, these results indicate that *Copia* was under stronger expansion than *Gypsy*.

Supplementary Note 3: Homoeologous Gene Retention Variation

We constructed an orthologous pan-genome comprising the 27 *B. oleracea* genomes. In total, we identified 57,137 orthologous gene families using OrthoFinder [10]. The number of orthologous families increased when adding additional genomes, approaching a plateau at n = 25 (**Supplementary Fig. 3a**), indicating that this pan-genome represents the majority of the *B. oleracea* gene repertoire. Of these orthologous families, 20,845 (36.5%), 8,516 (14.9%), 27,391 (47.9%), and 385 (0.7%) were considered as core, softcore, dispensable, and private gene families, respectively (**Supplementary Fig. 3b**), corresponding to a mean of 27,232 (52.1%), 11,274 (21.6%), 13,420 (25.7%), and 390 (0.8%) of core, softcore, dispensable, and private genes, respectively, in these 27 genomes (**Supplementary Fig. 3c**). There were 82% and 70% of the core and softcore genes, respectively, annotated with InterPro domains in the genome of T10, a wild *B. oleracea* genome with relatively high assembly continuity. These proportions were much higher than those for the dispensable and private genes (42% and 18%, respectively) (**Supplementary Fig. 3d**). Meanwhile, both the length and the number of CDS in the core and softcore genes were significantly higher than those in the dispensable and private genes (**Supplementary Figs. 3e** and **3f**). Besides, the K_a/K_s values were much higher in the dispensable/private genes than those of the core/softcore genes (**Supplementary Fig. 3g**), indicating higher conservation of function of the core/softcore genes and faster evolution of the dispensable/private genes.

We further investigated the copy-number variation of homoeologs among *B. oleracea* morphotypes. On average, ~60.25% of all genes had at least two homoeologs in the 27 genomes, similar to previous reports (Liu et al., 2014; Wang et al., 2011). We classified the syntenic genes into different groups according to the copy number of homoeologous genes (one, two or three). We found that the percentage of core genes from the category retaining three-copy homoeologs (51.5%) was considerably lower than the percentage from the categories of two-copy (61.2%) or single-copy (65.7%) homoeologs (**Fig. 2h**). Interestingly, we found many three-copy homoeologs that lost one or more copies in specific morphotypes. From the 3,744 three-copy homoeologs in

wild *B. oleracea* T10, 394 had specifically lost at least one copy in cabbage. These 394 genes were enriched in functions of cytoskeleton organization and actin binding. Another 543 of these wild *B. oleracea* three-copy homoeologs had lost at least one copy specifically in cauliflower and broccoli, and were enriched in functions of sulfate transport and metal ion binding (**Supplementary Tables 10 and 11**). The differential loss of homoeologs could contribute to the evolution of *B. oleracea* morphotypes through variation in gene copy dosage, which is associated with expression dosage.

Supplementary Note 4: SVs Introduce Expression Variation in Numerous Genes

We found that methylation was strongly associated with the suppressed expression of SV-genes. Remarkably, 75% of all SVs overlapped with TEs (**Supplementary Fig. 5c**). TEs are shown to have position effects; the transposition activity of TEs is silenced by methylation, which simultaneously silences the expression of nearby genes [11]. Whole-genome bisulfite sequencing in 16 of the 27 genomes revealed that 75% of all presence genotypes of SVs had weighted methylation ratio higher than 80%, illustrating that SVs are indeed highly methylated (**Supplementary Fig. 8a**). Of the 3,536 SVs that significantly suppressed gene expression (suppression SVs), 82% had significantly higher (P value < 0.05) methylation levels of sequences surrounding SV-genes, indicating that SVs suppress gene expression through methylation.

We used the nonredundant 50,153 SVs to construct an integrated graph-based genome with the T10 genome as a standard linear base reference (**Methods**). By mapping reads of 704 *B. oleracea* accessions to this graph-based genome, we revealed a total of 40,028 SVs in the population. The assessment indices (precision: 0.77, recall: 0.97, and F1 score: 0.86) for SV calling were comparable to those previously reported in soybean and rice [12,13]. We found that 34,484 SVs (86% of all SVs) were polymorphic in the 36 wild *B. oleracea* accessions, indicating that most SVs originated in wild *B. oleracea*. We then investigated the distribution of these SVs in the two independent domestication groups (LHL and AIL). In LHL and AIL, 96% and 94% of the 34,484 SVs were also polymorphic, respectively, showing the inheritance of these SVs in domesticated morphotypes. Of the 5,544 remaining SVs (14% of all SVs) that were not polymorphic in wild *B. oleracea*, 2,303 (42%) were polymorphic only in the LHL group, whereas 1,130 SVs (20%) were polymorphic only in the AIL group, indicating that these SVs arose during morphotype diversification of *B. oleracea* following the split of LHL and AIL.

Supplementary Note 5: Expression Alterations by SVs Associate with Morphotypes

We adopted the case-control genome-wide association study (GWAS) strategy [14,15] to identify SVs associated with different morphotypes of *B. oleracea* (**Methods**). Besides cauliflower/broccoli accessions, GWAS analysis was performed using cabbage accessions as the case group, which are characterized by the leafy heads The top 5% SV signals corresponded to 470 genes (**Supplementary Fig. 11a**), 361 of which were expressed. One-hundred-twenty-three and 112 SVs were negatively and positively associated with the expression of their SV-genes, respectively. Of the 112 promotion SVs, two SVs located closest to gene *BoKAN1*. *KAN1* regulates the leaf adaxial/abaxial polarity. Arabidopsis *kan1* mutants grow upward cup-shaped leaves compared to the basically flat leaves of wild-type plants, with slight down curvature [16-18]. SV1 (*P* value = 8.73×10^{-61}) locates 507 bp downstream of the translation stop site of *BoKAN1* and was introduced by a 970 bp TE (PIF/Harbinger) insertion (**Supplementary Fig. 11b**). This insertion is under strong negative selection in cabbage, being present in only four (1%) of the 289 cabbage accessions, compared to 59% (121/293) of all other accessions (**Supplementary Fig. 11c**). Another SV, SV2 (*P* value = 1.47×10^{-51}), located within the second intron of *BoKAN1* and was introduced by a 157 bp TE (Helitron) insertion. This insertion is also under negative selection in cabbage, present in 16% (47/297) of the cabbage accessions and 74% (246/332) of all other accessions. The two SVs form four haplotypes (**Supplementary Fig. 11d**). *BoKAN1* was significantly (*P* value = 3.60×10^{-7}) lower expressed in haplotypes 1 and 2, lacking the downstream SV1, than in accessions with SV1 present, while the expression was not associated with the intronic SV2 (**Supplementary Fig. 11e**). Yet another SV (*P* value = 3.69×10^{-91}) located closest to gene *BoACS4*. *ACS4* is the key regulatory enzyme in the biosynthesis of the plant hormone ethylene [19,20]. A previous study found that ethylene treatment suppresses the leaf heading process of cabbage through increasing internode lengths and that most ethylene biosynthetic genes are under negative selection in cabbage [21]. This SV of 1,671 bp located 2,186 bp upstream of the translation start site of *BoACS4* (**Supplementary**

Fig. 12a). Only three out of 292 (1%) cabbage accessions contained the insertion, while this insertion was present in 232 out of 314 accessions (74%) in all other accessions (**Supplementary Fig. 12b**). Expression of *BoACS4* in cabbage accessions lacking the insertion was significantly lower (P value = 1.90×10^{-14}) than in control accessions with the insertion (**Supplementary Fig. 12c**). In conclusion, the presence genotypes of the two SVs increased expression of their SV-genes *BoKAN1* and *BoACS4*, which are under strong negative selection in cabbage.

Supplementary Tables

Supplementary Tables 1, 2, 6, 7, 9-12, and 14-18 are provided in the spreadsheets of another excel file.

Supplementary Table 1. Summary of Illumina resequencing data for 415 *B. oleracea* accessions.

Supplementary Table 2. The information on the resequencing data of 704 *B. oleracea* accessions.

Supplementary Table 6. Summary of Bionano DLS molecules of five *B. oleracea* genomes.

Supplementary Table 7. Statistics of seven purged genomes with relatively high heterozygosity.

Supplementary Table 9. Summary of TE annotation for 27 *B. oleracea* genomes.

Supplementary Table 10. GO enrichment analysis of three-copy genes with homoeologous gene lost in cabbage.

Supplementary Table 11. GO enrichment analysis of three-copy genes with homoeologous gene lost in cauliflower/broccoli.

Supplementary Table 12. The 60 SVs that were manually checked by PacBio raw reads or Hi-C matrix.

Supplementary Table 14. The TF binding site prediction of the present sequence of promotion/suppression SVs.

Supplementary Table 15. The information of selected SVs for PCR amplification.

Supplementary Table 16. The results of SV-based eGWAS using 223 mRNA-seq datasets.

Supplementary Table 17. Summary of transcriptome sequencing data used for gene prediction.

Supplementary Table 18. Summary of whole genome bisulfite sequencing (methylation) data for 16 *B. oleracea* genomes.

Supplementary Table 3. Statistics of the long-read sequencing data for *B. oleracea* genomes.

Sample	Base Num (bp)	Coverage (X)	Mean length (bp)	N50 length (bp)	Platform
T02	137,098,795,148	235.97	13,474	21,008	Pacbio
T03	71,449,891,133	122.98	14,206	21,333	Nanopore
T04	88,285,325,194	151.95	20,588	28,925	Nanopore
T06	22,492,673,595	38.71	15,057	29,978	Nanopore
T07	76,127,574,931	131.03	22,176	32,632	Nanopore
T08	72,599,794,060	124.96	23,397	29,766	Nanopore
T09	97,892,717,083	168.49	23,992	32,899	Nanopore
T10	87,718,713,312	150.98	20,470	31,205	Nanopore
T11	77,377,889,062	133.18	22,278	30,743	Nanopore
T12	95,291,228,346	164.01	20,171	23,744	Nanopore
T13	81,016,616,982	139.44	16,977	21,715	Nanopore
T14	76,151,992,626	131.07	16,963	20,816	Nanopore
T15	79,254,764,136	136.41	20,733	26,222	Nanopore
T16	118,106,349,476	203.28	15,017	16,273	Nanopore
T17	97,027,765,446	167.00	20,897	26,102	Nanopore
T18	127,381,538,765	219.25	13,333	19,403	Pacbio
T19	96,538,805,858	166.16	19,814	26,484	Nanopore
T21	105,606,586,479	181.77	17,320	23,659	Nanopore
T24	42,699,837,370	73.49	5,851	13,044	Nanopore
T25	23,099,694,877	39.76	15,001	30,221	Nanopore
T26	38,463,983,201	66.20	9,359	18,525	Nanopore
T27	22,612,572,792	38.92	10,323	26,186	Nanopore

Supplementary Table 4. Summary of Illumina short-reads sequencing data for *B. oleracea* genomes.

Sample	Clean data (Gb)	Clean fold (×)
T02	137.82	237.21
T03	122.45	210.76
T04	134.57	231.62
T06	126.25	217.30
T07	158.59	272.96
T08	128.85	221.77
T09	128.94	221.93
T10	142.94	246.02
T11	299.14	514.87
T12	141.65	243.80
T13	140.94	242.58
T14	129.38	222.69
T15	228.24	392.84
T16	163.17	280.84
T17	148.85	256.20
T18	141.35	243.29
T19	191.62	329.81
T21	21.08	36.28
T24	140.67	242.12
T25	157.45	271.00
T26	151.54	260.83
T27	133.13	229.14

Supplementary Table 5. Summary of Hi-C sequencing data for *B. oleracea* genomes.

Sample	Data	Clean data (bp)	Clean fold (×)
T02	Hi-C	150,398,110,056	258.86
T03	Hi-C	150,460,371,622	258.97
T04	Hi-C	152,226,608,210	262.01
T06	Bionano	148,989,360,000	256.44
T07	Hi-C	150,890,913,058	259.71
T08	Hi-C	148,484,728,426	255.57
T09	Hi-C	155,904,679,784	268.34
T10	Hi-C	152,362,837,016	262.24
T11	Hi-C	152,637,782,624	262.72
T12	Hi-C	163,382,008,688	281.21
T13	Hi-C	151,497,158,590	260.75
T14	Hi-C	151,624,567,500	260.97
T15	Hi-C	178,931,493,834	307.97
T16	Hi-C	159,393,413,020	274.34
T17	Hi-C	150,239,614,450	258.59
T18	Hi-C	148,910,249,192	256.30
T19	Hi-C	150,728,600,446	259.43
T24	Bionano	110,568,570,000	190.31
T25	Bionano	96,467,170,000	166.04
T26	Bionano	105,621,640,000	181.79
T27	Bionano	155,614,870,000	267.84

Supplementary Table 8. BUSCO evaluation scores for the assembled genomes of *B. oleracea.*

Sample	BUSCO (%)
T02	99.2%
T03	99.1%
T04	99.0%
T06	97.1%
T07	98.8%
T08	99.3%
T09	99.3%
T10	99.4%
T11	99.4%
T12	99.3%
T13	99.2%
T14	99.2%
T15	99.0%
T16	98.5%
T17	98.4%
T18	99.1%
T19	99.4%
T21	98.6%
T24	97.5%
T25	97.0%
T26	97.1%
T27	97.4%

Supplementary Table 13. Summary of SV-genes.

Region	Total SV genes	Expressed SV genes	Promotion SV genes	Suppression SV genes	Association ratio (%)
CDS	722	374	96	214	82.89
intron	2,062	1,568	456	694	73.34
1.5 kb	3,628	3,357	1,082	1,192	67.74
3 kb	1,730	1,502	504	523	68.38
5 kb	1,375	1,147	370	414	68.35
10 kb	1,860	1,494	482	499	65.66

Supplementary Figures

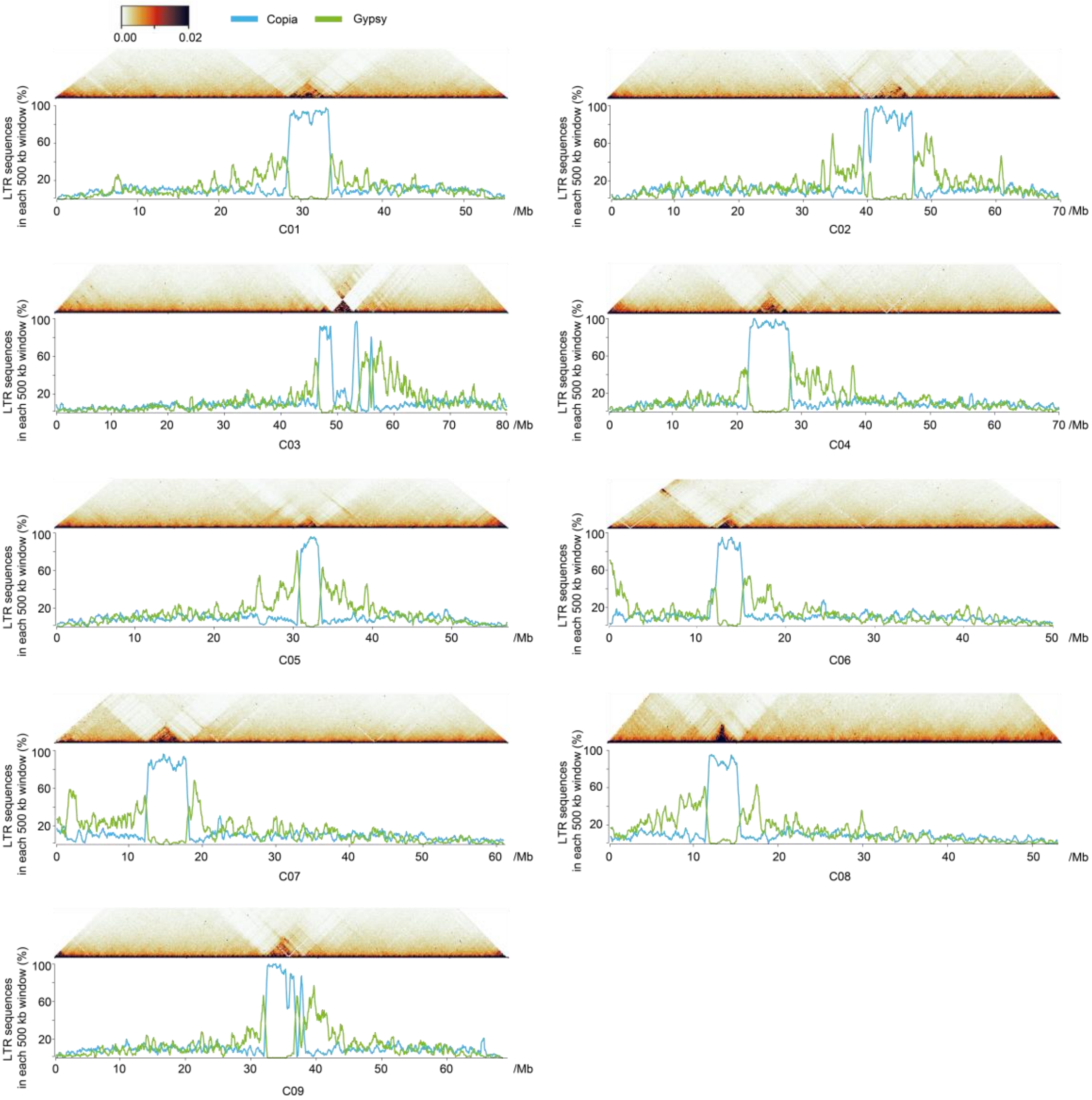

Supplementary Fig. 1. TAD prediction in nine chromosomes of the T10 genome. The heatmap shows the TAD prediction on nine chromosomes, in which the region colored in dark red denotes a TAD structure. The line charts below the heatmap shows the density of Copia and Gypsy LTRs within 500 kb sliding windows with 50 kb step size.

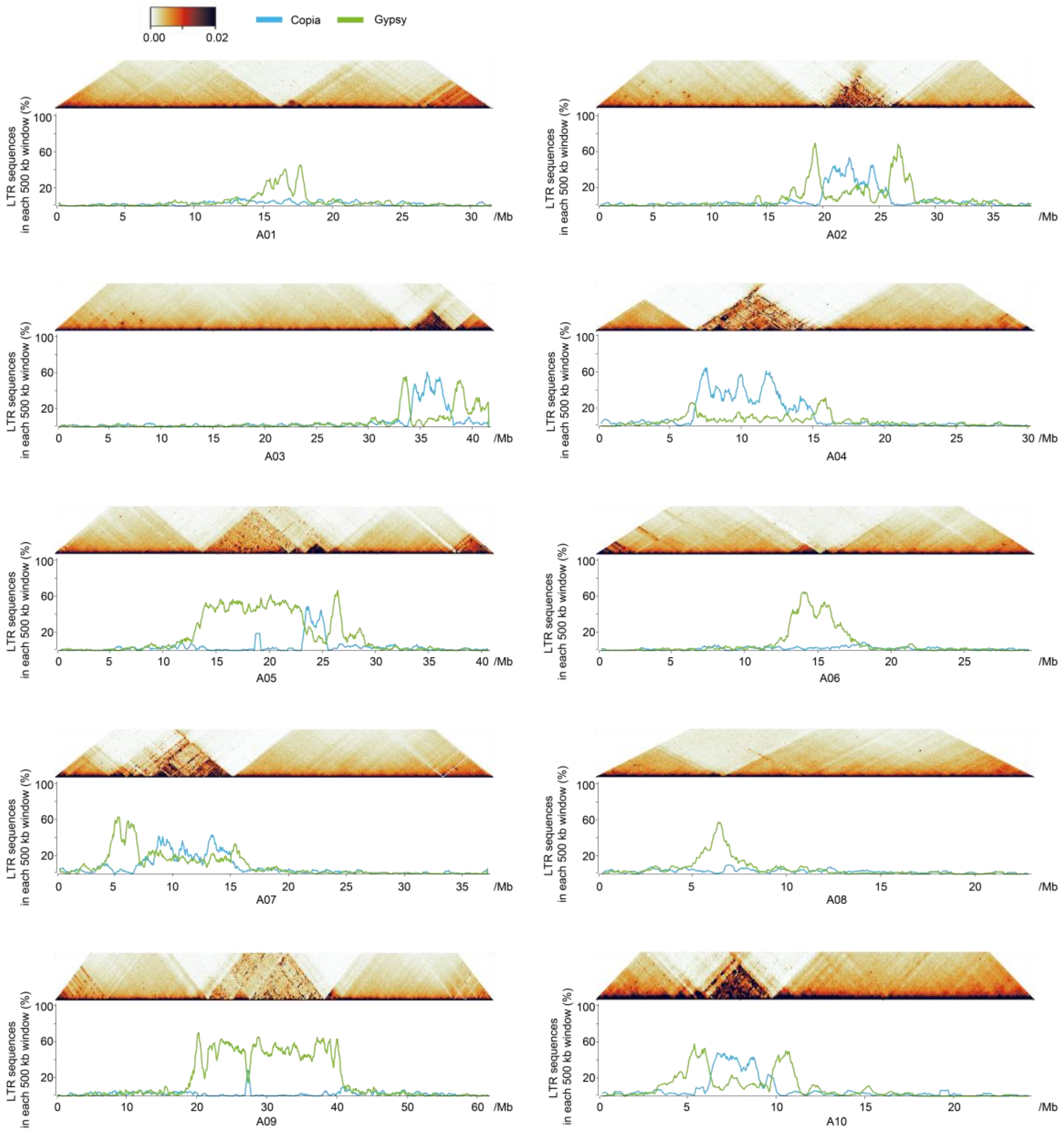

Supplementary Fig. 2. TAD prediction in ten chromosomes of *B. rapa* genome (A03 accession). The heatmap shows the TAD prediction on ten chromosomes, in which the region colored in dark red denotes a TAD structure. The line charts below the heatmap shows the density of Copia and Gypsy LTRs within 500 kb sliding windows with 50 kb step size.

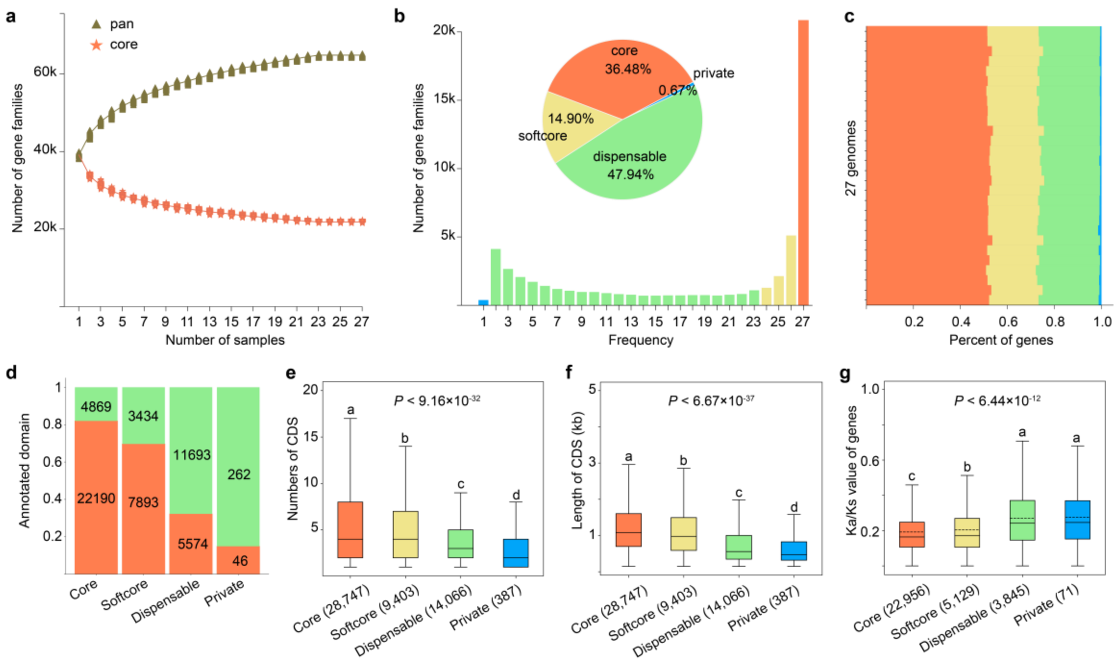

Supplementary Fig. 3. Characteristics of the orthologous pan-genome of *B. oleracea*. (**a**) The number of orthologous pan- and core-gene families in the 27 genomes. (**b**) Composition of the orthologous pan-genome. The histogram shows the frequency distribution of orthologous gene families shared by different numbers of genomes. The pie chart shows the proportion of different groups of orthologous gene families. (**c**) Percentage of different groups of orthologous gene families in each of the 27 genomes. (**d**) The proportion of orthologous gene families with InterPro domain annotations in different groups for the T10 genome. Orange and green bars represent orthologous gene families with and without InterPro domain annotations, respectively. (**e-g**) Boxplots of CDS number (**e**), CDS length (**f**) and K_a/K_s value (**g**) of orthologous core, softcore, dispensable, and private genes in the T10 genome. *P* values are determined by two-sided Student's *t*-test. The center line, box limits and whiskers represent median, first and third quartiles, 1.5× interquartile range, respectively. Different lowercase letters above the box plots represent significant differences ($P < 0.05$).

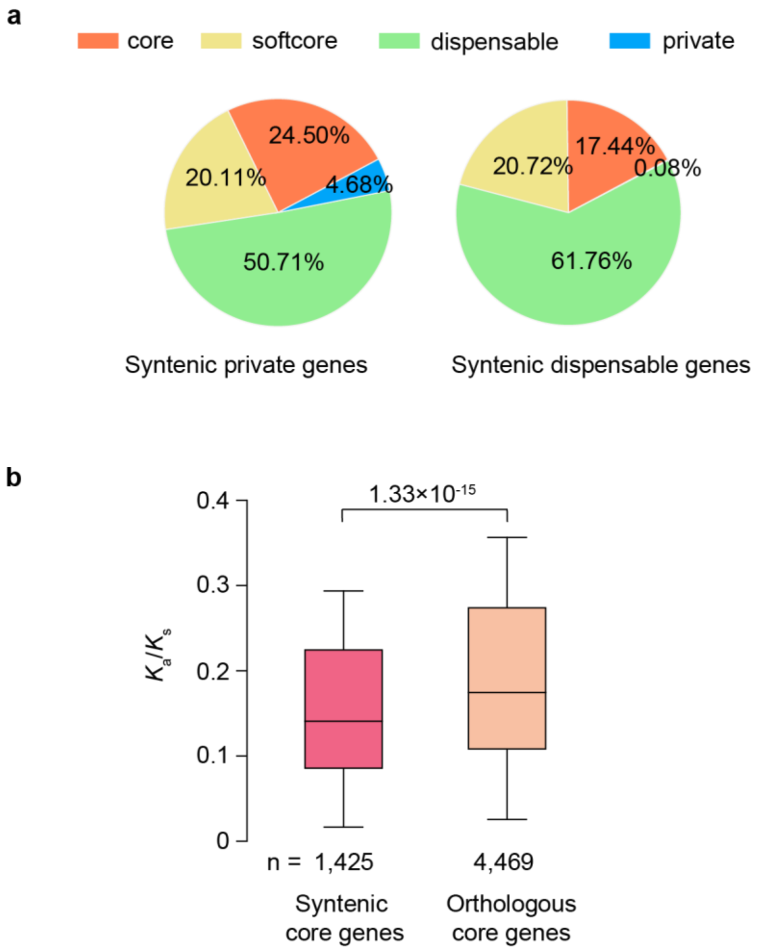

Supplementary Fig. 4. Comparison of the orthologous pan-genome and syntenic pan-genome. (**a**) The proportion of orthologous core, softcore, dispensable, and private genes in syntenic dispensable (left) and private (right) gene sets. (**b**) The comparison of K_a/K_s values between syntenic core genes and orthologous core genes. P values are determined by two-sided Student's t-test. The center line, box limits and whiskers represent median, first and third quartiles, 1.5× interquartile range, respectively.

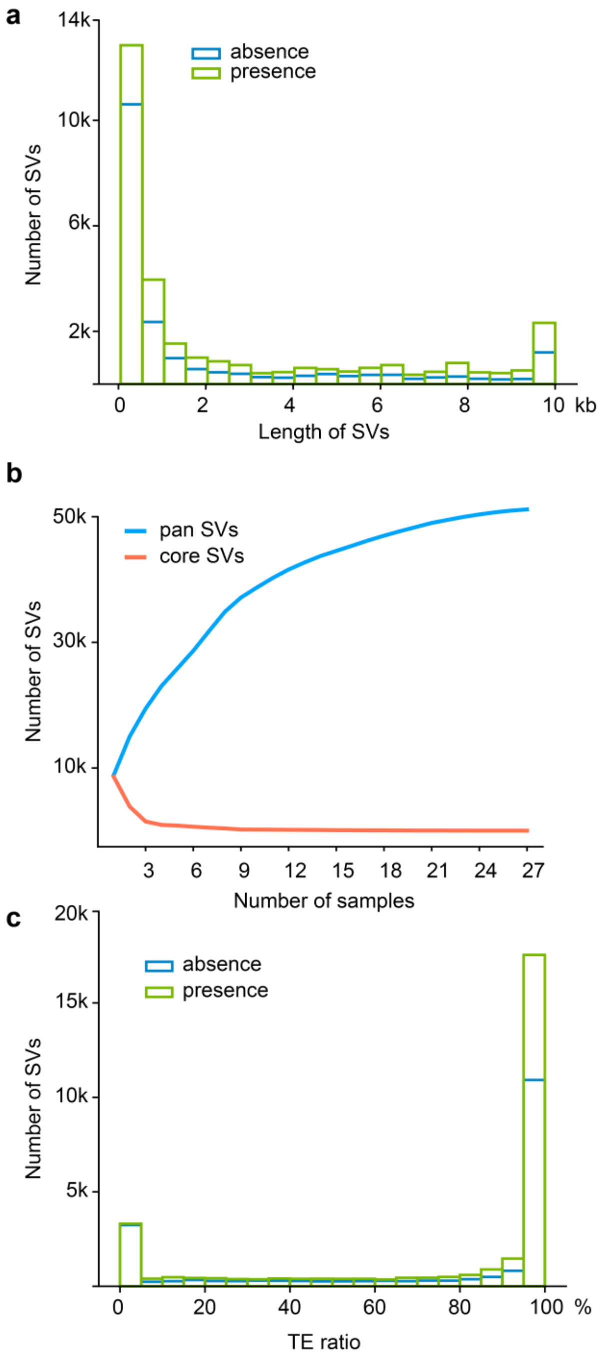

Supplementary Fig. 5. The characteristics of SVs in the 27 *B. oleracea* genomes. (**a**) Length distribution of presence/absence SVs. (**b**) The number of pan and core SVs with additional genomes being added. (**c**) The number of SVs that overlapped with different ratios of TE sequences.

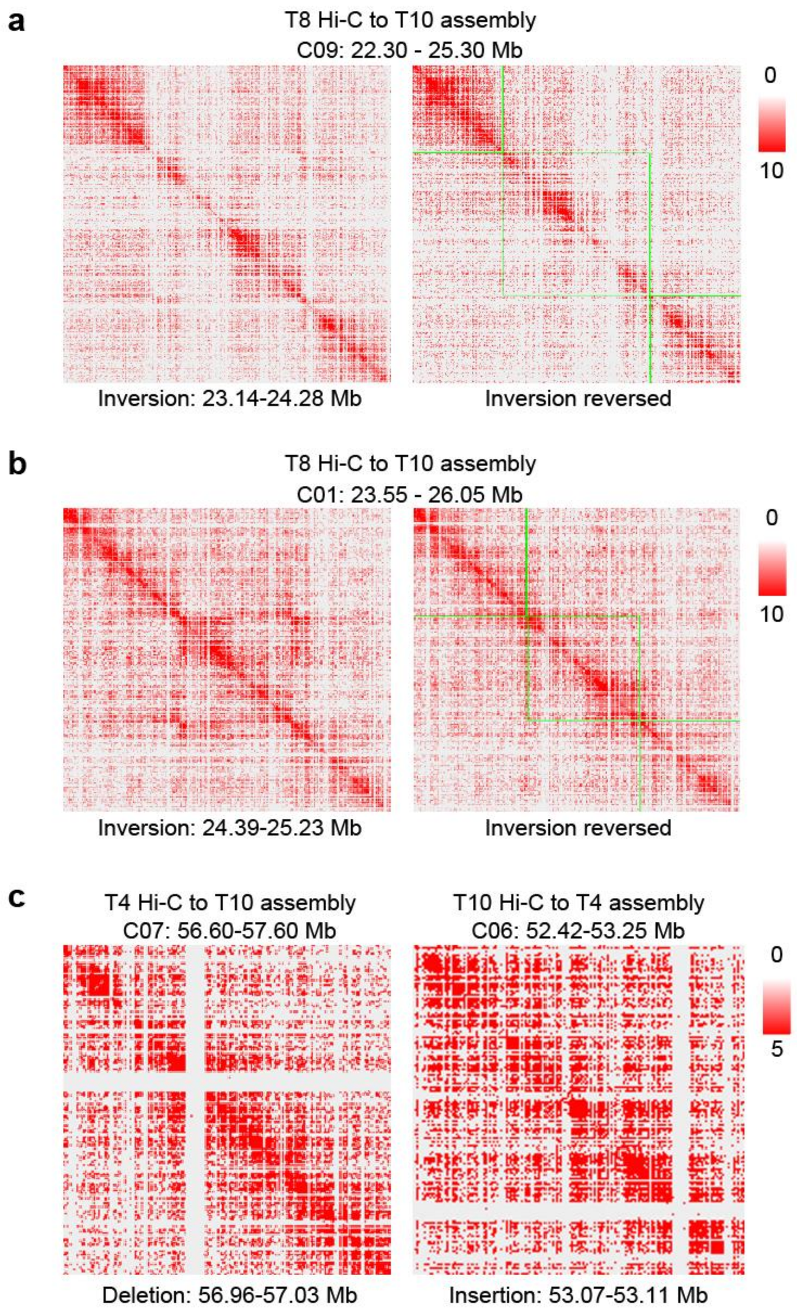

Supplementary Fig. 6. Examples of manual validation of SVs with lengths over 8 kb. (**a**) A randomly selected inversion in the T08 genome. The Hi-C reads of T08 were mapped to T10 genome at 5 kb resolution. The left graph shows the inversion region and the right graph shows the region with inversion being reversed. (**b**) A randomly selected inversion in T04 genome. The Hi-C reads of T04 were mapped to the T10 genome at 5 kb resolution. The left graph shows the inversion region and the right graph shows the region with inversion being reversed. (**c**) A randomly selected deletion in the T04 genome. The left panel: the Hi-C reads of T04 were mapped to T10 genome. The right panel: the Hi-C reads of T10 mapped to T04 genome.

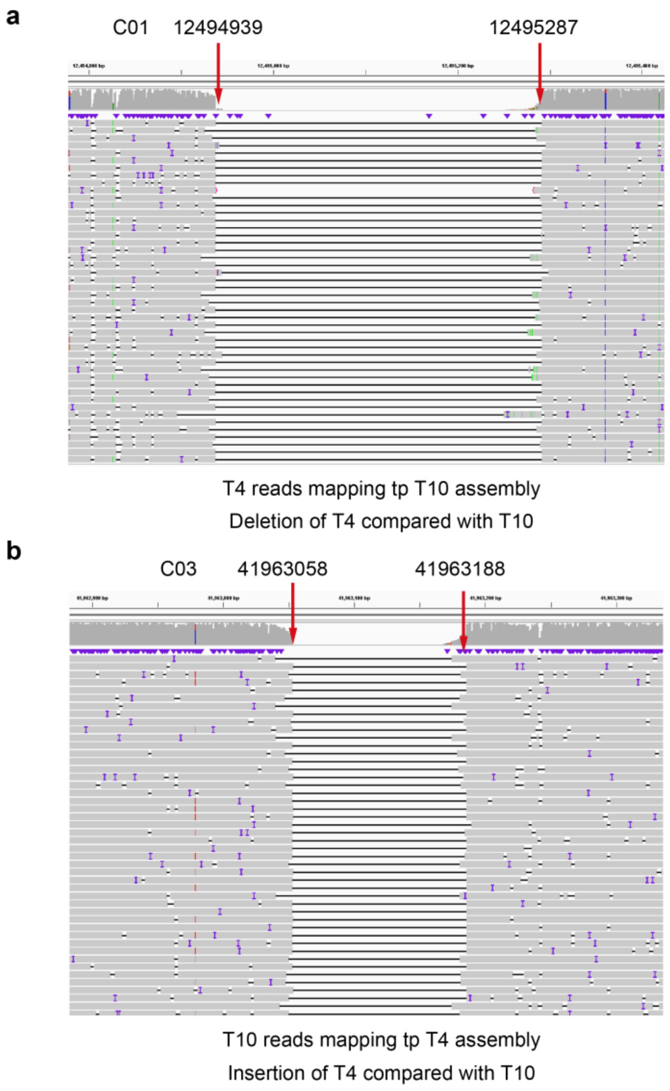

Supplementary Fig. 7. Examples of manual validation of SVs shorter than 8 kb. (**a**) A randomly selected deletion in the T04 genome, with the long-reads of T4 being mapped to the T10 genome. (**b**) A randomly selected insertion in the T04 genome, with the long-reads of T10 being mapped to the T04 genome. Red arrows indicate boundaries of the SV.

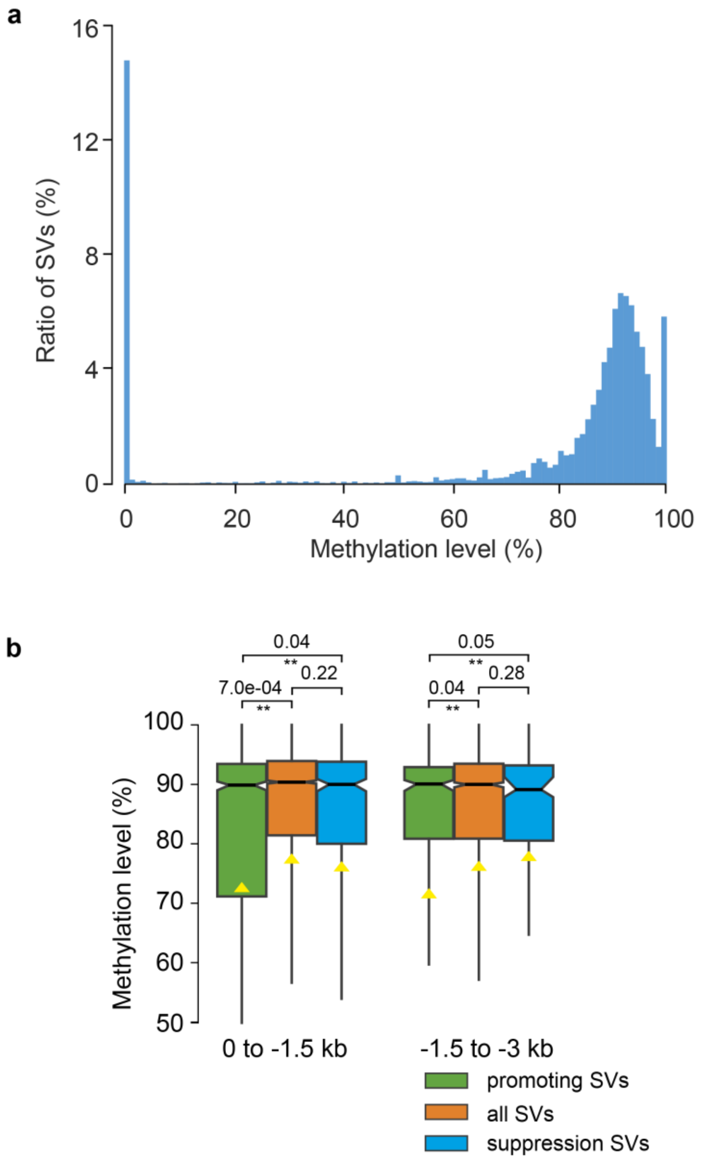

Supplementary Fig. 8. The methylation features of SVs. (**a**) The distribution of weighted methylation level for SVs. The *x* axis represents methylation level and *y* axis represents the corresponding SV ratio. (**b**) The comparison of methylation levels between promoting SVs, suppressing SVs and all SVs that are located till -1.5 kb (*n* = 484 *versus* 369 *versus* 2,794) or -3 kb (*n* = 153 *versus* 148 *versus* 1,391) of genes. Statistical tests were performed using 10,000 times permutation tests. Center line, triangle, box limits and whiskers represent the median, mean, first and third quartiles, and 1.5× interquartile range, respectively.

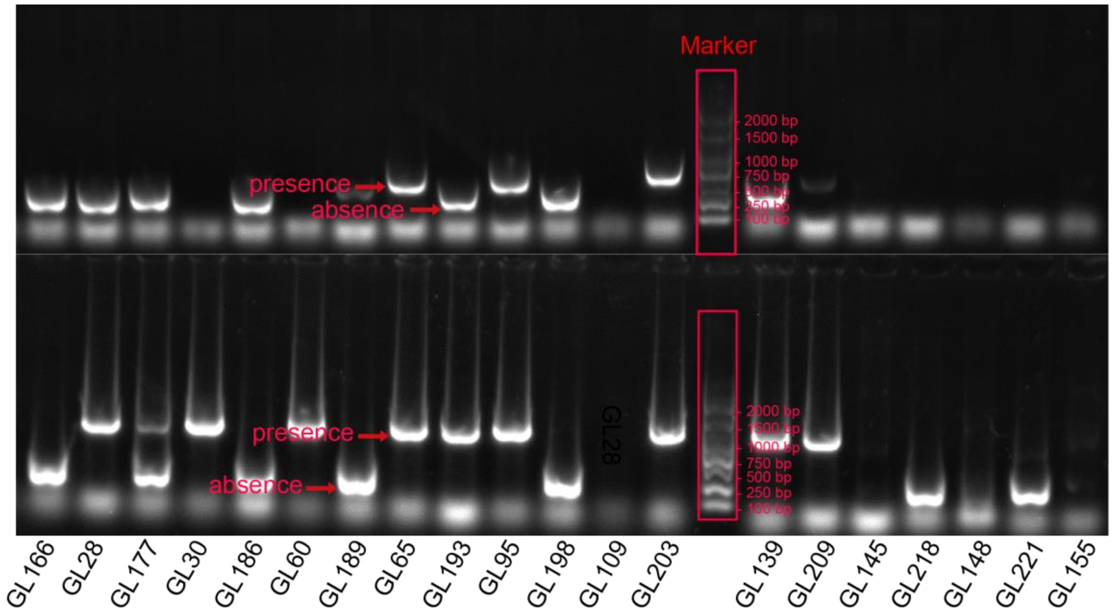

Supplementary Fig. 9. PCR amplification for SV validation. Two SVs were selected for validation. Different bands show the presence or absence of the two SVs amplified in accessions of *B. oleracea* (n = 20). The two SVs from top to bottom are a 227 bp insertion in the position of 16,867,020 bp in C05 and an 826 bp insertion in the position of 47,223,040 bp in C09.

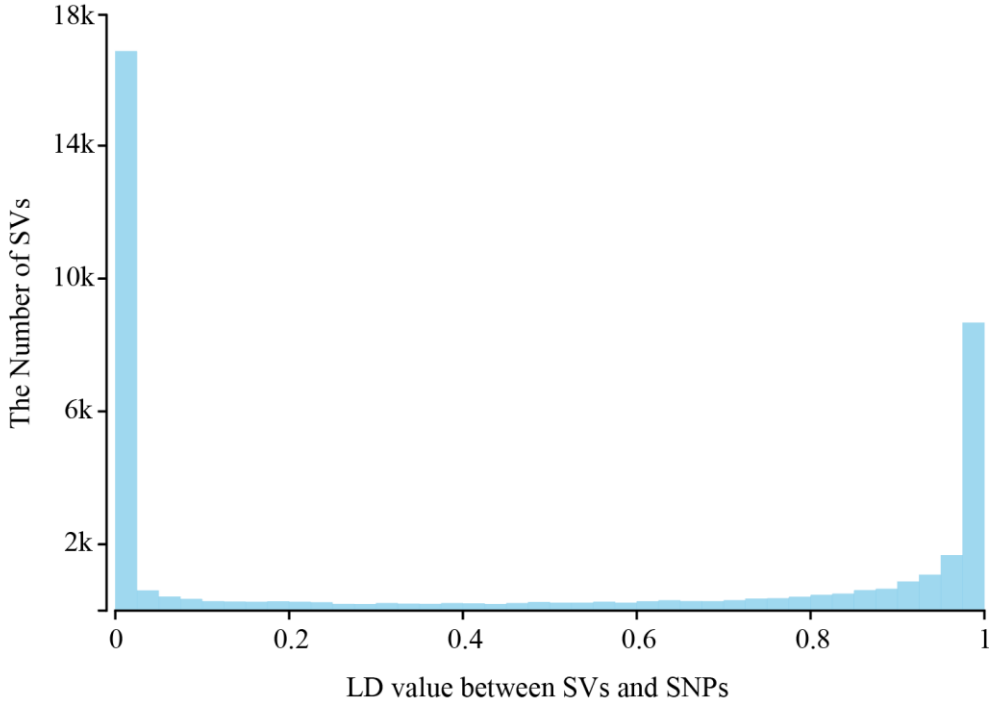

Supplementary Fig. 10. The distribution of Linkage disequilibrium (LD) values between SVs and SNPs (located within ±20 kb flanking regions of each SV).

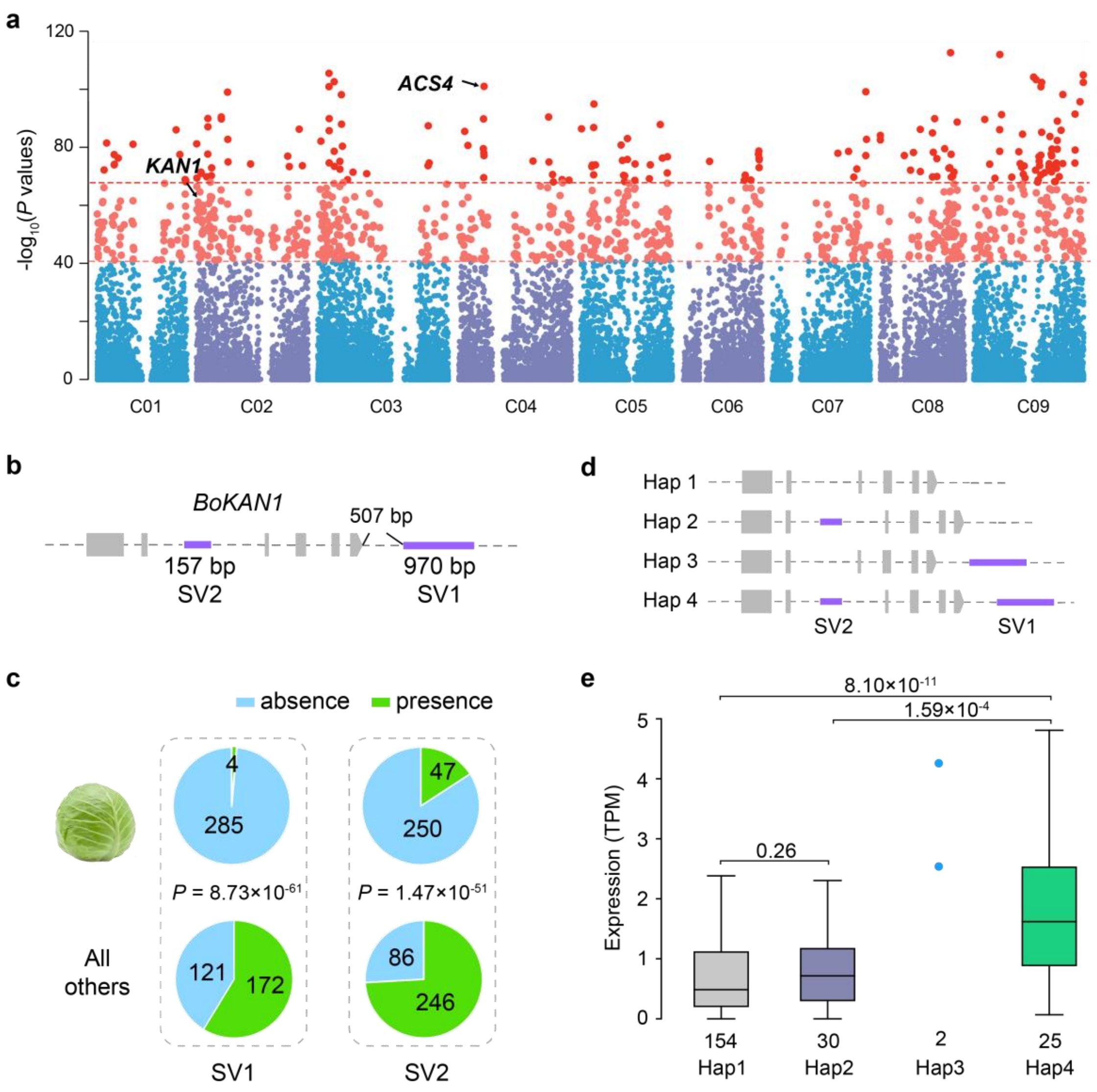

Supplementary Fig. 11. GWAS analysis of SVs associated with cabbage and the gene expression of four haplotypes of *BoKAN1*. (**a**) Manhattan-plot showing the genomic signals of SVs associated with cabbage (significance was calculated by two-tailed Fisher's exact test. A Bonferroni-corrected $P < 0.05$ was interpreted as significant). The light red dots indicate the top 5% *P* values and dark red dots show top 1% *P* values of SVs. (**b**) The two SVs associate with gene *BoKAN1*, with SV2 located at the second intron and SV1 located at +507 bp downstream of *BoKAN1*. (**c**) The numbers of accessions with different genotypes for the two SVs between the cabbages and all the other accessions. (**d**) The four haplotypes that are formed by different genotypes of the two SVs. (**e**) Boxplots show differences in the expression level of *BoKAN1* between the four haplotype groups (two-sided Student's *t*-test; centerline, median; box limits, first and third quartiles; whisker, 1.5× interquartile range).

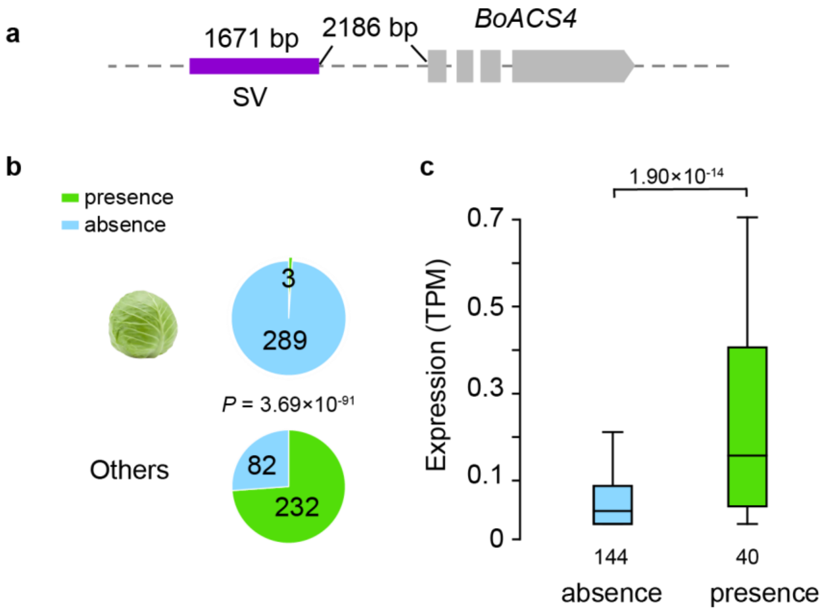

Supplementary Fig. 12. The SV under selection in cabbage was associated with gene *BoACS4*. (**a**) The SV was located in -2,186 bp upstream of *BoACS4*. (**b**) The number of accessions with different genotypes of SV for both cabbage and all other morphotypes. (**c**) Boxplot for expression levels of *BoACS4* comparing accessions with and without the SV (two-sided Student's *t*-test; center line, median; box limits, first and third quartiles; whiskers, 1.5× interquartile range).

Source Data Supplementary Fig. 9

Unprocessed gels for Supplementary Fig. 9

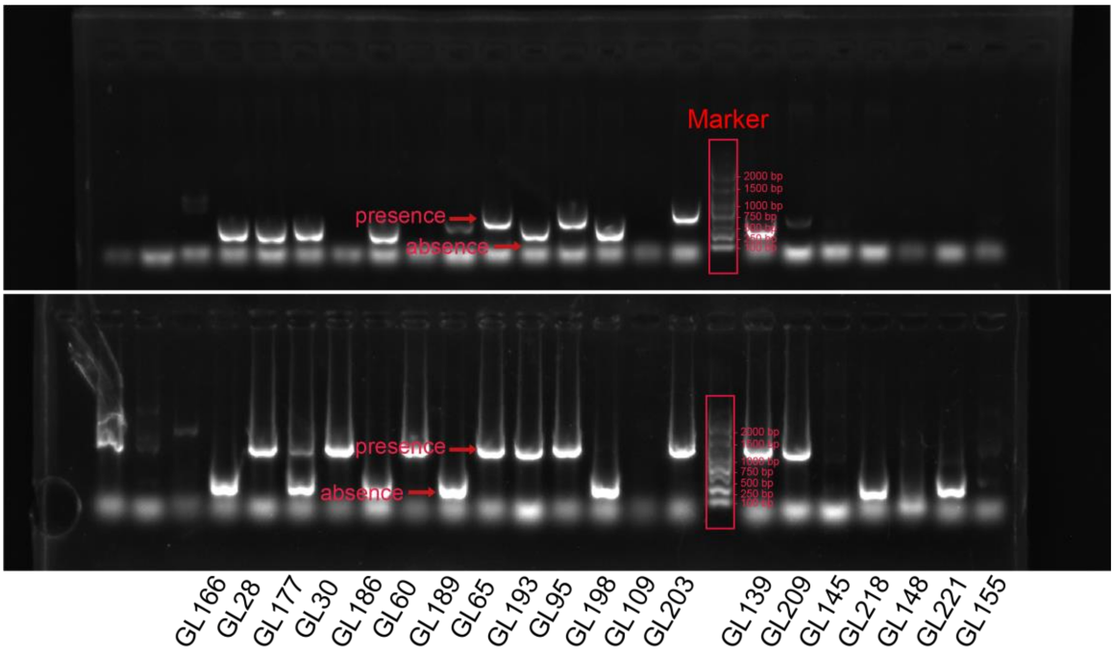

Supplementary References

1. Grob, S., Schmid, M.W. & Grossniklaus, U. Hi-C Analysis in Arabidopsis Identifies the KNOT, a Structure with Similarities to the flamenco Locus of Drosophila. *Molecular Cell* **55**, 678-693 (2014).
2. Murray, M.G. & Thompson, W.F. Rapid isolation of high molecular weight plant DNA. *Nucleic Acids Res* **8**, 4321-5 (1980).
3. Allen, G.C., Flores-Vergara, M.A., Krasynanski, S., Kumar, S. & Thompson, W.F. A modified protocol for rapid DNA isolation from plant tissues using cetyltrimethylammonium bromide. *Nat Protoc* **1**, 2320-5 (2006).
4. Shen, Y. *et al.* Global dissection of alternative splicing in paleopolyploid soybean. *Plant Cell* **26**, 996-1008 (2014).
5. Lv, H. *et al.* A high-quality reference genome for cabbage obtained with SMRT reveals novel genomic features and evolutionary characteristics. *Sci Rep* **10**, 12394 (2020).
6. Cai, X. *et al.* Improved Brassica oleracea JZS assembly reveals significant changing of LTR-RT dynamics in different morphotypes. *Theor Appl Genet* **133**, 3187-3199 (2020).
7. Belser, C. *et al.* Chromosome-scale assemblies of plant genomes using nanopore long reads and optical maps. *Nat Plants* **4**, 879-887 (2018).
8. Guo, N. *et al.* Genome sequencing sheds light on the contribution of structural variants to Brassica oleracea diversification. *BMC Biol* **19**, 93 (2021).
9. Cai, X. *et al.* Impacts of allopolyploidization and structural variation on intraspecific diversification in Brassica rapa. *Genome Biol* **22**, 166 (2021).
10. Emms, D.M. & Kelly, S. OrthoFinder: phylogenetic orthology inference for comparative genomics. *Genome Biol* **20**, 238 (2019).
11. Hollister, J.D. & Gaut, B.S. Epigenetic silencing of transposable elements: a trade-off between reduced transposition and deleterious effects on neighboring gene expression. *Genome Res* **19**, 1419-28 (2009).
12. Liu, Y. *et al.* Pan-Genome of Wild and Cultivated Soybeans. *Cell* **182**, 162-176 e13 (2020).
13. Qin, P. *et al.* Pan-genome analysis of 33 genetically diverse rice accessions reveals hidden genomic variations. *Cell* **184**, 3542-3558 e16 (2021).
14. Balding, D.J. A tutorial on statistical methods for population association studies. *Nat Rev Genet* **7**, 781-91 (2006).
15. Wellcome Trust Case Control, C. Genome-wide association study of 14,000 cases of seven common diseases and 3,000 shared controls. *Nature* **447**, 661-78 (2007).
16. Alvarez, J.P., Furumizu, C., Efroni, I., Eshed, Y. & Bowman, J.L. Active suppression of a leaf meristem orchestrates determinate leaf growth. *Elife* **5**(2016).
17. Mach, J. Getting in Shape? Leaves work it out with KANADI1. *Plant Cell* **26**, 4 (2014).
18. Kerstetter, R.A., Bollman, K., Taylor, R.A., Bomblies, K. & Poethig, R.S. KANADI regulates organ polarity in Arabidopsis. *Nature* **411**, 706-9 (2001).
19. Dong, Z. *et al.* Abscisic Acid Antagonizes Ethylene Production through the ABI4-Mediated Transcriptional Repression of ACS4 and ACS8 in Arabidopsis. *Mol Plant* **9**, 126-135 (2016).
20. Abel, S., Nguyen, M.D., Chow, W. & Theologis, A. ACS4, a primary indoleacetic acid-responsive gene encoding 1-aminocyclopropane-1-carboxylate synthase in Arabidopsis thaliana. Structural characterization, expression in Escherichia coli, and expression characteristics in

response to auxin [corrected]. *J Biol Chem* **270**, 19093-9 (1995).

21. Zhang, K. *et al.* A cluster of transcripts identifies a transition stage initiating leafy head growth in heading morphotypes of Brassica. *Plant J* **110**, 688-706 (2022).
